# Complex patients – an academism or reality?

**DOI:** 10.3325/cmj.2023.64.61

**Published:** 2023-02

**Authors:** Aleksandar Džakula, Karmen Lončarek, Dorja Vočanec

**Affiliations:** 1 Department of Social Medicine and Organization of Health Care, Andrija Štampar School of Public Health, University of Zagreb School of Medicine, Zagreb, Croatia; 2 Center for Integrated and Palliative Care, University of Rijeka, Faculty of Medicine, Rijeka, Croatia

Conant-Ashby theorem: “Every good regulator of a system must be a model of that system.”

In the article Lost in Thought: The Limits of the Human Mind and the Future of Medicine published in *The New England Journal of Medicine* in 2017, the authors deal with a new challenge in medicine – complexity. They state the overall problem: *“Medical thinking has become vastly more complex, mirroring changes in our patients, our health care system, and medical science. The complexity of medicine now exceeds the capacity of the human mind.*” (1).

Does this mean that the complexity of reasoning and decision-making will directly (negatively) affect the quality of health care? Moreover, is the complexity of modern medicine the only complexity to which we should pay attention in health care?

## The return of humanity and complexity

For a long time, the only terms associated with patient complexity were comorbidity and multimorbidity. However, the term “complex patient” was formed after the development of some new concepts in other sciences and professions: complexity science, social determinants of health, and patient-centered and people-centered health care. After more than 20 years, we have a clearly defined concept of the “complex patient,” as well as the problems that arise from complexity in providing care (2-4).

However, are we ready to recognize these concepts in practice? To what extent are we aware of the importance of understanding the complexity, and more importantly, are we capable of developing health care that effectively responds to it – comprehensive, integrated, and aligned with the 24/7/365 model of care? Modern medicine was founded on scientific research and its main creed is evidence-based medicine. Biological, bio-medical, and clinical research is the basis of good results and continuous development of medicine. However, the limitations that arise in the use of modern medicine (both at the individual and community level!) leave many patients without adequate care. This is why we increasingly talk about new inequalities in health and the inadequate availability of health care. Modern medicine contributes to these problems, because technological and bio-medical approaches do not take into account the social determinants of health, which undoubtedly play a role in the overall outcome of care.

## Social determinants and health

In the first half of the 20th century, before the great momentum of medical technologies and related innovations, the social determinants of health were the key targets of state interventions. Still today, medical care accounts for only 10%-20% of modifiable contributors to healthy outcomes for a population (5).

**Figure 1:**
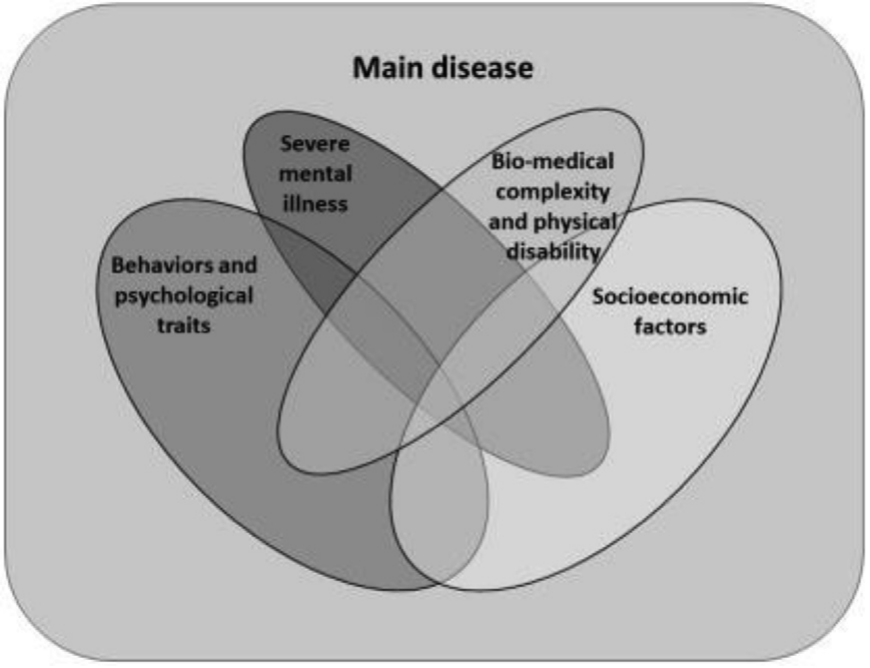
Determinants of patients' complexity.

At that time, social determinants were primarily related to population health and interventions in large communities plagued by poor hygiene conditions, infectious diseases, or severe addictions. Today, however, we are witnessing a revival of social medicine, but on different premises. Namely, in the last twenty years, especially through the concept of patient-centered medicine, social determinants have been closely related to each individual patient. This is because it is crucial to provide appropriate care that meets the specific needs of individuals, especially complex patients who require case management, which is now widely recognized as an essential component of quality health care (6). Having said that, case management should incorporate not only disease management but should arise from a wider bio-psycho-social model of care (Figure 1). Since medical practitioners have practically and conceptually separated themselves from society and social issues, these efforts should include other professions, and target social determinants of health on both the clinical and community level (7). An additional challenge is that interventions aimed at integrating medical and social practice are long-term and difficult to measure because they take place in a reality of many interdependent relationships and processes (8).

## More of the same: difficult or complex?

Generators of the increasing complexity of care are aging, comorbidities, and the increasing possibilities of modern medicine. However, this is only a partial explanation. New technologies that prolong life also facilitate care provision. Furthermore, the high proportion of the elderly population, which by biological necessity suffers from comorbidity and multimorbidity, is not a new problem. It has existed for several decades, and during that time the health care systems have been able to adapt. For this reason, nowadays the term “complexity” is increasingly used for situations that go beyond the domain of somatic health and affect the areas of mental health and social care. Even the smallest problem in these dimensions can dramatically worsen the patient's condition or make it difficult to achieve desired care outcomes. Small limitations related to social determinants or a mental health problem can quickly cause a dramatic increase in complexity.

In short, more diseases or more engaged health care resources to which modern medicine contributes do not mean greater complexity. A problem that requires a greater “quantity” of solutions is more difficult, but not necessarily more complex. Complexity arises from the interdependence of multiple interconnected elements. When it comes to the patient, the complexity arises from his or her capacity as a person to receive available help, which is influenced by social determinants and the mental health state of the person (9-11).

## Even the rich cry

Social determinants of health usually mean social deprivation of individuals, the population, or the community. Poverty and scarcity cause reduced opportunities for providing care (and thus care management), which in turn worsens both the health status and social determinants, thus creating a vicious circle. From the health care point of view, we see this vicious circle as complexity. The share of complex patients is higher in poor societies, where finding solutions is more difficult as well, at least from the perspective of developed countries. However, health inequalities and disadvantaged population groups persist in Organisation for Economic Co-operation and Development countries as well (12). So, a developed society has an ethical responsibility to take care of the disadvantaged and not to deepen the gap within the population through basic settings of the care system.

Therefore, even in developed countries complexity due to social determinants remains a major issue, with individuals receiving less and less support from their own social networks as a result of the transition and structural change of community and family. In societies of lower socio-economic development, where there is still some form of sustained family that can provide care and security to the patient, the complexity is of a somewhat different character.

Another concern in Western countries is the realization that there is a limit above which more money and resources do not lead to better health outcomes. This is again connected to the rising complexity in attending to a certain patient, where the success and overall outcome result from coordinated and holistic care processes rather than from providing perfect care in each segment. These notions, translated into the language of policy makers and health economists, are found in the emerging concepts such as value-based health care, strategic purchasing, and bundled payments, all aimed at societal well-being rather than at just health outcomes (13).

## Conclusion

Is there something we can finally conclude? Definitely! The concept of a “complex patient” is well defined at the research and academic level – therefore, there should be no great wanderings in the recognition or categorization of complex patients.

Policy documents clearly recognize complex patients and the need to align the care systems according to this complexity as a priority for the EU and most countries globally. In addition, health care workers face complex patients as a challenge in everyday practice. Decision-makers are well aware that total costs for complex patients are continuously increasing.

However, there is a wide gap between knowledge and interventions, which causes patients’ suffering and problems in the functioning of the health care system. These problems are reflected in a series of related phenomena. First, on the supply side, the health care and social care systems are fragmented, and their separation continuously worsens. Second, on the demand side, the scope of the individuals’ needs and their number are constantly growing. On top of that, the number of individuals living in single households is increasing, with families and communities becoming weaker and less connected. Finally, there is a global shortage of (professional) workforce in the care sector, which is constantly worsening. All this poses great challenges and the need for a paradigm change in proposing possible solutions and interventions.

If we will summarize the conclusions and translate them into the language of SWOT analysis – we have to invest, mobilize, and implement harm reduction interventions in the care of complex patients. And do that immediately and at full sail!
